# Increasing incidence of mucormycosis in a large Spanish hospital from 2007 to 2015: Epidemiology and microbiological characterization of the isolates

**DOI:** 10.1371/journal.pone.0179136

**Published:** 2017-06-07

**Authors:** Jesús Guinea, Pilar Escribano, Antonio Vena, Patricia Muñoz, María del Carmen Martínez-Jiménez, Belén Padilla, Emilio Bouza

**Affiliations:** 1 Clinical Microbiology and Infectious Diseases Department, Hospital General Universitario Gregorio Marañón, Universidad Complutense de Madrid, Madrid, Spain; 2 Instituto de Investigación Sanitaria Gregorio Marañón, Madrid, Spain; 3 CIBER de Enfermedades Respiratorias (CIBERES CB06/06/0058), Madrid, Spain; 4 Medicine Department, School of Medicine, Universidad Complutense de Madrid, Madrid, Spain; Leibniz Institute for Natural Products Research and Infection Biology- Hans Knoell Institute, GERMANY

## Abstract

We studied 19 cases of proven/probable mucormycosis diagnosed from 2007 to 2015 in our hospital and assessed the microbiological characteristics of the isolates. We recorded the incidence of mucormycosis and clinical and microbiological data of infected patients. Isolates were identified to molecular level and tested for their antifungal susceptibility to azoles, amphotericin B, and liposomal amphotericin B according to the CLSI M-38 A2 procedure. The incidence of mucormycosis in cases/100,000 hospital admissions during 2007–2015 increased significantly with respect to that reported in 1988–2006 (3.3 vs. 1.2; *P*<0.05). Patients mainly had hematological malignancies (52.6%) and/or trauma/surgical wounds (52.6%) and had received antifungal agents before the diagnosis of mucormycosis in 68% of cases. Diagnosis was by isolation (n = 17/19) and/or direct staining (n = 17/18) of Mucorales fungi in clinical samples. Identification was by panfungal PCR in patients with negative results in culture and in direct staining. The microorganisms identified were *Lichtheimia* spp. (42%), *Rhizopus* spp. (21%), *Cunninghamella bertholletiae* (16%), and others (21%). Liposomal amphotericin B was always more active than the other drugs against all the microorganisms except *C*. *bertholletiae*. All patients received antifungal treatment with 1 or more antifungal agents, mainly liposomal amphotericin B (17/19). Mortality was 47.4%, although this was significantly lower in the 11 patients in whom debridement was performed (18% vs. 87.5%) (*P* = 0.015). The incidence of mucormycosis has risen in recent years. The proportion of cases with soft tissue involvement was high, and *Lichtheimia* was the most frequently involved species. The highest antifungal activity was observed with liposomal amphotericin B.

## Introduction

Mucormycosis is a rapidly progressive and severe sporadic invasive disease caused mainly by the species *Mucor*, *Rhizopus*, *Rhizomucor*, and *Lichtheimia* (formerly *Absidia*) [1]. The incidence of mucormycosis has increased in recent years. The role of overuse of voriconazole in this increase is under debate because few studies have focused on this matter [2–4], and those that did were not specifically designed to assess the problem [5]. Another recent issue is that of a potential shift in the underlying conditions of patients with mucormycosis from diabetes mellitus [1, 2], to hematological malignancies [6]. Consequently, it is necessary to study changes in the incidence of mucormycosis and analyze its epidemiology in large general hospitals.

PCR-based procedures can detect and accurately identify Mucorales fungi in clinical samples when cultures are negative and thus improve our understanding of the epidemiology of mucormycosis [7–12]. Unfortunately, molecular procedures are not widely available in the day-to-day practice of the microbiology laboratory, and diagnosis is based on fungal isolation and/or direct examination in clinical samples. Moreover, antifungal susceptibility testing is necessary to understand susceptibility patterns, although most data come from morphologically identified isolates.

The aims of the present study were to analyze the epidemiology and incidence of mucormycosis from 2007 to 2015 and to perform in-depth microbiological characterization of isolates (molecular detection in clinical samples, molecular identification of isolates, and antifungal susceptibility testing).

## Materials and methods

### Hospital description

Hospital General Universitario Gregorio Marañón serves a population of approximately 715,000 inhabitants in the city of Madrid, Spain and cares for patients at high risk of mucormycosis, such as those admitted to medical and surgical ICUs, patients with hematological malignancies, solid organ transplant recipients, patients with trauma or surgical wounds, and patients with diabetes mellitus. A total of 570,949 hospital admissions were recorded during the study period.

### Patients and microbiological diagnosis

From January 2007 to December 2015, a total of 19 patients fulfilled the diagnosis of proven (n = 12) or probable (n = 7) mucormycosis according to the revised definitions of invasive fungal disease of the European Organization for Research and Treatment of Cancer/Mycosis Study Group (EORTC/MSG) [13], with the following modification: case no. 15 was also considered probable and the diagnosis was made by detection of Mucorales using PCR with normally sterile samples in the absence of histopathology findings and fungal isolation.

Laboratory-based diagnostics included conventional procedures (fungal culture, direct fungal stain, and histopathology), and nonconventional procedures (panfungal PCR detection in samples with a request for a microbiology work-up) [7]. For patients with proven/probable mucormycosis, we collected demographic, clinical, and microbiological data and outcome. Categorical variables were described and compared using the chi-square or Fisher exact test. The incidence of the infection was calculated and compared with that reported previously from 1988 to 2006 using standard binomial methods [14].

### Molecular identification and antifungal susceptibility testing

We studied morphologically identified Mucorales isolates (n = 42) from 15 of the 19 patients with proven/probable mucormycosis (cultures were negative in 2 patients, and isolates were not available in another 2 patients) and an additional 34 nonsignificant isolates.

The molecular identification of the 76 isolates was performed by sequencing the ITS1-5.8S-ITS2 region of the ribosomal genes [15]. A BLAST search of all the sequences was performed to identify the isolates. Reference sequences retrieved from GenBank were included to construct a phylogenetic tree and confirm molecular identification. Antifungal susceptibility to conventional amphotericin B (Sigma-Aldrich, Madrid, Spain), liposomal amphotericin B (Gilead Ltd, Madrid, Spain), itraconazole (Janssen Pharmaceutical Research and Development, Madrid, Spain), voriconazole (Pfizer Pharmaceutical Group, New York, NY, USA), and posaconazole (Merck & Co., Inc., Rahway, NJ, USA) was determined using the CLSI M38-A2 procedure [16]. The stock of liposomal amphotericin B was obtained after reconstitution of an intravenous vial according to the manufacturer’s instructions. The final concentration of the antifungal agents in the plates ranged from 0.003 μg/mL to 8 μg/mL. All the inoculated trays were incubated at 35°C for 24 hours, and the MIC was visually defined as the concentration that completely inhibited fungal growth. Antifungal susceptibility to the 5 drugs was compared using the Kruskal-Wallis test for independent samples.

### Ethical considerations

This study (protocol no. 323/14) was approved by the Ethics Committee of Hospital Gregorio Marañón [CEIC-A1]). The need for informed consent was waived owing to the retrospective design of the study.

## Results

### Incidence of the infection and description of patients

From January 2007 to December 2015, a total of 19 patients were diagnosed with proven/probable mucormycosis, ie, an incidence of 3.3 cases (range, 0 to 6.2) per 100,000 hospital admissions.

Data for patients with proven/probable mucormycosis are summarized in Table 1. Most were male (n = 15, 79%), and their mean age was 56.42±18.31 years. At diagnosis, 42% were admitted to oncology-hematology wards and 37% to ICUs or other wards (21%). The main predisposing conditions for the infection were hematological malignancies (52.6%) and skin trauma or surgical wounds (52.6%). Ten patients (52.6%) had skin and soft tissue infection, and 8 (42%) patients (6 of whom had underlying hematological-oncological conditions) had lower respiratory tract involvement; the remaining patient had rhino-sinusal involvement. Patients with lung infection were immunosuppressed, whereas only 6 of the 10 patients with superficial infections were immunocompromised; superficial infections affected mainly catheter drainage/insertion sites or surgical wounds (Table 1).

**Table 1:** Clinical description of the 19 patients with mucormycosis collected during the study period.

Patient	Year	Age/Sex	Underlying condition	Organs affected (samples)	Mycological criteria				Species found	Radiology	Antifungal treatment		Outcome
					Culture	Rapid stain	PCR	Histology			At diagnosis ^****^	Targeted	
1 ^*^	2007	77 / M	COPD, cirrhosis, myeloproliferative syndrome (treatment with hydroxyurea)	Lung (bronchial secretions)	+	+	ND	ND	*L*. *ramosa* + *A*. *fumigatus*	Chest x-ray (pleural effusion)	No	L-AmB + VOR	Poor
**2**	**2007**	**41 / M**	**Acute myeloid leukemia, chemotherapy, neutropenia**	**Lung (BAL, biopsy)**	**+**	**+**	**ND**	**+**	***L*. *ramosa ***	**CT scan (halo sign)**	**L-AmB** **(empirical)**	**L-AmB switched to POS** **+ surgery**	**Alive**
**3**	**2007**	**61 / F**	**Myelodysplastic syndrome, chemotherapy, neutropenia**	**Drainage insertion site (skin biopsy)**	**+**	**+**	**ND**	**+**	***R*. *pusillus***	**ND**	No	**L-AmB** **+ POS** **+ surgery**	**Alive**
**4**	**2008**	**68 / M**	**Heart transplant, surgery**	**Mediastinal wound after transplant (skin wound and sternum biopsy)**	**+**	**+**	**ND**	**+**	***M*. *circinelloides***	**ND**	**No**	**L-AmB** **+ POS** **+ surgery**	**Alive**
5	2008	48 / M	Lymphoblastic lymphoma, chemotherapy, neutropenia	Lung (sputum)	+	ND	+	ND	*L*. *corymbifera*	CT scan (bilateral infiltrates, nodules)	CAS (empirical)	CAS + VOR	Poor
**6**	**2008**	**75 / M**	**Surgery on the left arm for skin graft collection**	**Surgical wound (skin)**	**+**	**+**	**ND**	**+**	***L*. *corymbifera ***	**ND**	**FLU** **(empirical)**	**L-AmB** **+ surgery**	**Alive**
**7**	**2009**	**80 / M**	**Surgery on the right arm for skin graft collection**	**Surgical wound (skin)**	**+**	**+**	**ND**	**+**	***L*. *corymbifera ***	**ND**	**CAS** **(empirical)**	**L-AmB** **+ surgery**	**Poor**
**8**	**2009**	**61 / M**	**Lymphoblastic acute leukemia, chemotherapy, neutropenia, trauma**	**Area of puncture for bone marrow collection, left buttock (skin wound)**	**+**	**+**	**-**	**+**	***L*. *ramosa***	**ND**	**POS** **(prophylaxis)**	**L-AmB** **+ surgery**	**Alive**
**9** ^*^	**2009**	**34 / M**	**Hodgkin lymphoma, autologous HSCT, chemotherapy, neutropenia,**	**Skin, left subcostal flank (biopsy)**	**+**	**+**	**ND**	**+**	***R*. *pusillus*** ***+ A*. *fumigatus***	**ND**	**CAS + VOR (aspergillosis treatment)**	**L-AmB**	**Poor**
10	2009	47 / M	Hodgkin lymphoma, chemotherapy, neutropenia,	Lung (BAL, bronchial secretions)	+	+	-	ND	*C*. *bertholletiae*	CT scan (bilateral infiltrates)	No	VOR switched to L-AmB + POS	Poor
11	2010	44 / M	Acute myeloid leukemia, allogeneic HSCT, GVHD	Lung (bronchial secretions)	+	+	-	ND	*R*. *arrhizus*	CT scan (infiltrates, cavities)	VOR (prophylaxis)	L-AmB + POS	Alive
12 ^*^	2011	61 / F	Metastatic cervical carcinoma, chemotherapy, corticosteroids	Lung (sputum)	+	+	-	ND	*C*. *bertholletiae* *+ A*. *fumigatus*	CT scan (cavities)	FLU (prophylaxis)	L-AmB + POS + MYC	Poor
**13**	**2012**	**60 / M**	**Liver transplantation, tacrolimus, corticosteroids, surgery**	**Surgical wound after transplantation (skin)**	**+**	**+**	**-**	**+**	***M*. *circinelloides***	**ND**	**No**	**L-AmB** **+ surgery**	**Alive**
**14**	**2012**	**76 / M**	**Insect bite**	**Insect bite site (skin wound)**	**+**	**+**	**+**	**+**	***S*. *vasiformis***	**ND**	**No**	**L-AmB** **+ POS** **+ surgery**	**Alive**
15 ^**^	2012	4 / F	Lymphoblastic acute leukemia, corticosteroids, neutropenia	Lung (pleural fluid)	-	-	+	ND	*C*. *bertholletiae*	CT scan (consolidation, pleural effusion)	MYC (prophylaxis)	L-AmB	Poor
16	2014	49 / M	Diffuse large B-cell lymphoma, haploidentical HSCT, neutropenia	Lung and sinus (BAL, bronchial secretions, sputum)	+	+	+	ND	*L*. *ramosa*	CT scan (bilateral infiltrates, pleural effusion, sinus affectation)	MYC (prophylaxis)	L-AmB + POS	Poor
**17** ^*^	**2015**	**60 / M**	**Interstitial lung disease, corticosteroids**	**Catheter insertion (skin biopsy)**	**+**	**+**	**+**	**+**	***R*. *arrhizus*** ***+ A*. *fumigatus***	**ND**	**VOR** **(treatment of aspergillosis)**	**L-AmB** **+ surgery**	**Poor**
**18**	**2015**	**72 / F**	**Surgery,** **squamous cell carcinoma**	**Surgical wound (skin biopsy)**	**+**	**+**	**-**	**+**	***L*. *ramosa***	**ND**	**No**	**L-AmB** **+ surgery**	**Alive**
**19**	**2015**	**54 / M**	**Diabetes**	**Paranasal sinus (biopsy)**	**-**	**+**	**-**	**+**	**Unable** ^***^	**CT scan (sinus destruction and osteomyelitis)**	**MYC** **(empirical)**	**L-AmB** **+ POS** **+ surgery**	**Alive**

**F**, female; **M**, male; **L-AmB**, liposomal amphotericin B; **FLU**, fluconazole; **CAS**, caspofungin; **VOR**, voriconazole; **POS**, posaconazole; **MYC**, micafungin; **COPD**, chronic obstructive pulmonary disease; **HSCT**, hematopoietic stem cell transplantation; **GVHD**, graft versus host disease; **ND,** not done; **BAL,** bronchoalveolar lavage

Cases of proven mucormycosis are in boldface

^*^ Patients 1, 9, 12, and 17 had an additional diagnosis of probable invasive pulmonary aspergillosis.

^**^ Cultures of samples from the patient were negative, and species were identified using panfungal PCR on direct clinical samples.

^***^ Cultures of samples and panfungal PCR detection were negative for Mucorales in this patient.

^****^ Antifungal agent received at diagnosis of mucormycosis.

The following antifungal agents were administered in the month previous to the diagnosis in 13 patients (68%): fluconazole (n = 7), caspofungin (n = 3), voriconazole (n = 2), micafungin (n = 3), liposomal amphotericin B (n = 1), and posaconazole (n = 2). Antifungal treatment at diagnosis is shown in Table 1. As for management of the infection, patients received antifungal treatment with one or more antifungal agents, mainly liposomal amphotericin B (18/19, 95%). Mortality was 47.4%, although this was significantly lower in the 11 patients in whom therapeutic debridement was performed (18% vs. 87.5%) (*P* = 0.015). The mortality of the patients with lung infection was higher (70%) than that of patients with superficial infection (30%), although the differences did not reach statistical significance.

### Diagnosis of mucormycosis

Diagnosis was mainly by isolation of Mucorales (n = 17/19) and/or direct staining (n = 17/18) in clinical samples (Table 1). Skin involvement mainly indicated proven infection, probably owing to the availability of samples for both microbiology and histopathology work-ups. In contrast, histopathology data were not available for patients with lung involvement, and infections were classified as probable based on both the presence of Mucorales in lower respiratory tract samples and compatible radiological findings (Table 1). Panfungal PCR detection was performed on samples from 12 patients and identified the species in a patient with negative culture and calcofluor stain results (patient no. 15) and in 2 patients in whom the stored isolates were not available for molecular identification (patients no. 5 and 14). The PCR results were concordant with those of species identification performed on pure cultured isolates (n = 2) or were false negatives (n = 7).

A concomitant diagnosis of probable invasive pulmonary aspergillosis was established in 4 patients (2 with probable pulmonary mucormycosis and 2 with proven skin mucormycosis) (Table 1); mucormycosis and aspergillosis were diagnosed almost simultaneously (± 1 day), with the exception of 1 case (patient no. 17), in whom the diagnosis of mucormycosis was established 14 days later.

### Etiological agents and antifungal susceptibility testing

The species detected in the 19 patients were *Lichtheimia ramosa* (n = 5), *Lichtheimia corymbifera* (n = 3), *Cunninghamella bertholletiae* (n = 3), *Rhizomucor pusillus* (n = 2), *Mucor circinelloides* (n = 2), *Rhizopus arrhizus* (n = 2), *Saksenaea vasiformis* (n = 1), and unknown (patient no. 19) (Table 1). Only 1 species was detected in patients with multiple isolates. We did not find any association between the species detected and the source of the clinical sample or underlying conditions, although the low number of cases precluded conclusive results.

The antifungal susceptibility of the isolates is shown in Table 2. Liposomal amphotericin B showed the highest *in vitro* activity (mean MIC, 0.16 mg/L), followed by posaconazole (mean MIC, 0.38 mg/L), conventional amphotericin B (mean MIC, 0.76 mg/L), itraconazole (mean MIC, 2.13 mg/L), and voriconazole (mean MIC, 14.08 mg/L) (*P*<0.001). Liposomal amphotericin B showed higher activity than amphotericin B in all isolates except *C*. *bertholletiae* (*P*<0.05).

**Table 2:** Antifungal susceptibility of the 76 isolates studied.

Species	No. ^1^	Geometric mean MIC and range (in μg/ml)				
		AmB	L-AmB	VOR	ITC	POS
*Lichtheimia ramosa*	15 / 8	0.49	0.009	>8	1.33	0.26
*Lichtheimia corymbifera*	6 / 2	1.10	0.11	>8	1	0.25
*Rhizomucor pusillus*	4 / 3	0.37	0.09	>8	0.9	0.45
*Mucor circinelloides*	9 / 5	1.1	0.09	>8	>8	1.22
*Cunninghamella bertholletiae*	6 / 0	2	>8	>8	1	0.5
*Rhizopus arrhizus*	2 / 11	0.85	0.10	9.90	2.48	0.22
*Rhizopus microsporus*	0 / 5	0.76	0.66	8	1.15	0.33
Overall	76	0.76 (0.062–2)	0.16 (0.031->8)	14.08 (2->8)	2.13 (0.5->8)	0.38 (0.062–2)

^1^ Number of significant/nonsignificant isolates. Antifungal susceptibility testing could not be performed on isolates from 4 patients because the isolates were either not available (patients no. 5 and 14) or the cultures were negative (patients no. 15 and 19

## Discussion

Our study shows that the incidence of mucormycosis has risen in the last 10 years in our institution. The frequency of pulmonary mucormycosis is higher than before, the possibility of surgical intervention is lower, and mortality remains very high. *Lichtheimia* spp. were the most common cause of infection, and a significant number of episodes may have been polyfungal.

Multicenter studies [17], and single-center studies [18, 19], have reported an increasing incidence of mucormycosis over time. We previously reported 12 patients diagnosed from 1988 to 2006 and estimated an incidence of 1.2 cases/100,000 admissions at our institution [14]. This finding is in line with that reported in a multicenter study conducted in Spain in 2005 (0.62 cases/100,000 admissions) [20]. However, the incidence during 2007–2015 increased significantly (3.3 cases/100,000 admissions, *P*<0.05). Some authors state that the use of voriconazole prophylaxis can explain this increase, although the issue is still under debate [12]; in fact, only 10% of patients in the present study had previously received voriconazole, although many of them had received other agents with poor anti-Mucorales activity.

In the large series by Roden and colleagues, the most commonly involved sites were the sinuses (39%), lungs (24%), and skin (19%) [1]. In the more recent series by Skiada and colleagues, hematological disorders were the predisposing conditions in half of the cases, and diabetes mellitus (17%) and trauma (17%) were less common [21]. Our series includes cases collected as recently as 2015, and half of the patients had hematological disorders. A high proportion of patients had skin and soft tissue involvement (52%), and a lower percentage of diabetes mellitus and rhino-cerebral involvement than in previous reports [1, 21, 22], including one from our institution [14].

Microbiological diagnosis was mainly by isolation of Mucorales and/or positive direct staining in clinical samples (89%). These findings are line with those of previous studies [21]. Complementary application of fungal culture, direct examination, and panfungal PCR procedures increased sensitivity, as previously reported [22]. Panfungal PCR was introduced in our laboratory in 2009 and proved useful for species identification in 3 patients, although it yielded a considerable number of false negatives, probably owing to the limited amount of sample processed.

Our cases were most frequently caused by *Lichtheimia* spp. (42%), followed by *Rhizopus* spp. (21%), *C*. *bertholletiae* (16%), and other species (21%). Geographic area may be relevant, as shown by previous reports from Australia and Europe [19, 21–24]. Morphological identification of *Lichtheimia* spp. commonly leads to misidentification of the 4 pathogenic species for humans [25–28], and 5 of our cases were *L*. *ramosa*. *S*. *vasiformis* infected an immunocompetent patient after an insect bite on the scalp (a well-known route of acquisition) [29]; panfungal PCR detection was particularly useful in this case, as the isolate sporulated poorly and morphological examination was insufficient. We found pulmonary mucormycosis caused by *C*. *bertholletiae* in 3 patients with hematological cancer or solid tumors, one of whom (patient no. 15), was diagnosed only by panfungal PCR. Interestingly, although *C*. *bertholletiae* isolates were systematically less susceptible to the agents tested than other species, they were particularly refractory to liposomal amphotericin B. Poor results with amphotericin B have been reported in an experimental model and in patients [2, 30]. Of note, the 3 patients infected by *C*. *bertholletiae* died. Future research should be performed on the epidemiology and treatment of *Cunninghamella* infections.

The recent ESCMID guidelines recommend surgical debridement in addition to immediate first-line antifungal treatment with liposomal or lipid-complex amphotericin B for adults and children; posaconazole may be used as salvage therapy [31]. Most of the patients in the present study received liposomal amphotericin B (90%). Surgical debridement, which was performed in 58%, improved outcome. Our impression is that a more aggressive surgical approach, particularly for localized pulmonary lesions, even in patients with hematologic cancer, could reduce the very high mortality recorded in this population, as shown in patients with aspergillosis in France [32]. The mortality of our series exactly matched that reported by Skiada et al and Kennedy et al [21, 22].

The main strengths of our study are that our hospital has operated an alert system for the diagnosis mucormycosis since almost 30 years ago, with the result that missing cases should not be relevant in this series. In addition, use of molecular tools for diagnosis and species identification provided accurate epidemiological data. Finally, we studied the incidence of the infection and compared it with that previously reported in the hospital. Our study is limited by the low number of cases reported.

In conclusion, we report a recent increase in the incidence of mucormycosis in our hospital. The proportion of cases with soft tissue involvement was high, and *Lichtheimia* spp. was the most commonly involved species. Our findings could serve as the basis for future studies on the management of patients in similar institutions. Further analysis of local epidemiology could provide more substantial data.
