# Correction: Increasing incidence of mucormycosis in a large Spanish hospital from 2007 to 2015: Epidemiology and microbiological characterization of the isolates

**DOI:** 10.1371/journal.pone.0229347

**Published:** 2020-02-12

**Authors:** Jesús Guinea, Pilar Escribano, Antonio Vena, Patricia Muñoz, María del Carmen Martínez-Jiménez, Belén Padilla, Emilio Bouza

There are errors in the Funding statement. The correct Funding statement is as follows: This study was supported by a research grant from Gilead and by grants ref. CM-SANTANDER (GR3/2014; group 920200), PI14/00740, MSI15/00115 FIS (FIS, Instituto de Salud Carlos III; Plan Nacional de I+D+I 2013–2016) and by the Spanish Network for Research in Infectious Diseases (REIPI RD12/0015). The study was cofunded by the European Regional Development Fund (FEDER) `A way of making Europe.’ PE (CPI15/00115) and JG (CPII15/00006) hold a Miguel Servet contract; LJMZ (FI12/00265) is supported by FIS. The funders had no role in the study design, data collection and analysis, decision to publish, or preparation of the manuscript.
